# Alcohol Withdrawal Syndrome as a Precipitating Factor of Takotsubo Cardiomyopathy on a Background of Wernicke’s Encephalopathy

**DOI:** 10.7759/cureus.27288

**Published:** 2022-07-26

**Authors:** Inês N Costa, Joana S Reis, Ana O Monteiro, Catarina Fernandes, Manuela Dias

**Affiliations:** 1 Medical Oncology, Centro Hospitalar Universitário de São João, Porto, PRT; 2 Internal Medicine, Centro Hospitalar Universitário de São João, Porto, PRT

**Keywords:** catecholamine excess, thiamine deficiency, alcohol withdrawal syndrome, wernicke encephalopathy, takotsubo cardioyopathy

## Abstract

Takotsubo cardiomyopathy (TC) is a rare entity defined by a temporary, reversible left ventricular systolic abnormality similar to myocardial infarction in the absence of coronary artery disease. Alcohol withdrawal syndrome (AWS) can result from a marked decrease or sudden cessation of alcohol consumption and seems to be related to excess catecholamines that induce a cardiomyotoxic effect. Wernicke's encephalopathy (WE), mostly associated with alcoholism, is caused by a deficiency in vitamin B1 (thiamine) and is severe if not supplemented in a timely manner. We present a complex case of a patient with the simultaneous presentation of two rare conditions, takotsubo cardiomyopathy, and Wernicke's encephalopathy, precipitated by an alcohol withdrawal syndrome.

## Introduction

Takotsubo cardiomyopathy (TC) is a rare heart disease with a prevalence of 1.0-2.5%. Its presentation can mimic acute coronary syndrome (ACS) without angiographic evidence of vessel occlusion. The most typical echocardiographic sign is apical ballooning involving the left ventricle, which results from apical dyskinesia or akinesia with basal hyperkinesia. This is a complicated pathology and still little known, although evidence does show catecholamines in excess precipitated by emotional or physical stressors may be responsible for TC. Systolic dysfunction may spontaneously reverse if the patient receives adequate hemodynamic support [1].

There are some reported cases of TC associated with alcohol withdrawal syndrome (AWS) [2]. Sudden cessation of alcohol consumption induces increased adrenergic activity with excessive release of catecholamines and, consequently, cardiotoxicity [1].

Wernicke's encephalopathy (WE) is often associated with alcoholism and has an estimated prevalence of 0.4-2.8% [3]. It is caused by a deficiency of vitamin B1 (thiamine). The classic triad of symptoms (altered mental status, ataxia, and oculomotor abnormality) is not always present, which requires a high degree of suspicion to immediately initiate thiamine treatment and prevent progression to Korsakoff syndrome, which has an even higher mortality rate [4].

## Case presentation

A 51-year-old female was admitted to the emergency department for hallucinations, generalized tremors, and difficulty in walking independently. She had a history of 10-pack-year smoking and a long duration of excessive consumption of alcohol (90 g/daily). The sudden suspension of alcohol consumption in the week prior to admission. She is a rare frequenter of health services and has no regular compliance with medication. She lived in housing with poor hygienic and sanitary conditions.

Vital signs included a 36.0 ºC temperature, a 76/minute pulse rate, blood pressure at 132/85 mmHg, a 16/minute respiratory rate, and 100% saturation of oxygen in room air. She is a thin patient showing signs of psychomotor agitation. The speech was mostly incoherent. She had visual and tactile hallucinations and gait ataxia. No flapping tremor was observed. The cardiovascular examination showed S1 and S2 rhythmic, without audible murmurs. Respiratory auscultation was normal. The abdomen was soft, painless, without hepatomegaly or ascites, and normal bowel sounds. No peripheral oedema.

She presented metabolic alterations such as hypokalaemia (3.0 mEq/L) and hypomagnesemia (1.28 mEq/L). In the analytical study aimed at cardiac function, high sensitivity troponin I was found to be high at 76.0 ng/L (normal range <14) and elevated brain natriuretic peptide at 679.6 pg/mL (normal range <100).

The remaining analytical studies showed normal liver function. Lumbar puncture was not performed because, in addition to not having an increase in infection markers, the patient had no other symptoms such as fever or neck stiffness.

A brain CT scan showed cerebellar and supratentorial atrophy, predominantly fronto-temporo-parietal cortical. It was assumed to be an AWS. The patient was medicated with benzodiazepine and given fluid therapy with 5% dextrose. As she showed confusion and gait ataxia, the possibility of concomitant WE was also considered. In our hospital, it is not possible to measure thiamine levels, but, given the suspicion, it was decided to immediately start parenteral administration of thiamine 200 mg in combination with complex B vitamins.

**Figure 1:**
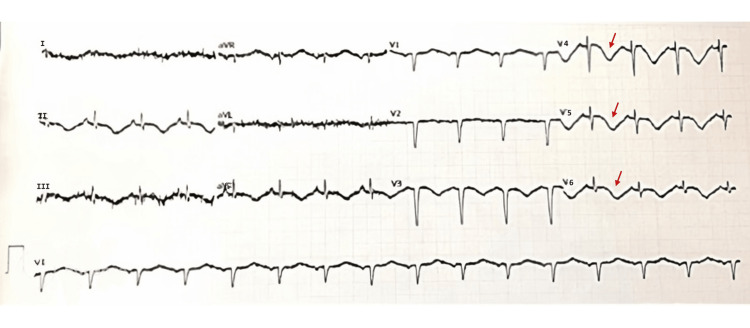
Electrocardiogram Sinus rhythm, heart rate of 92 bpm, Q waves in the anterior wall, inversion of the T wave in the lateral wall (arrows) and increase in the QTc interval (620 ms).

**Figure 2:**
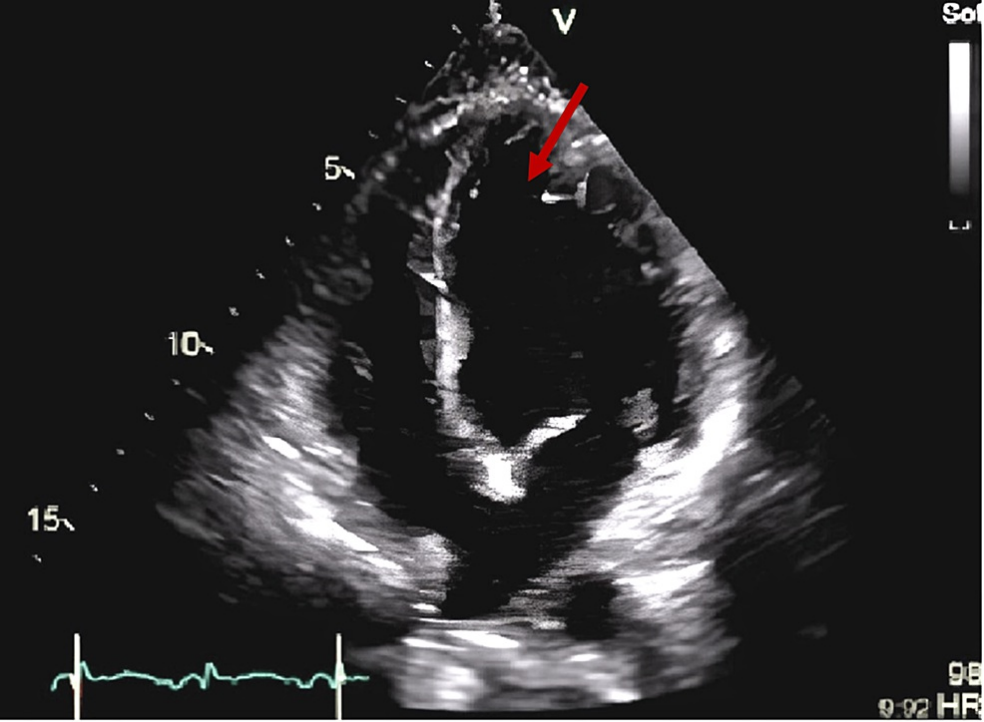
Transthoracic echocardiography Moderately to severely compromised systolic left ventricular function (ejection fraction of 32%), with mid-distal akinesia (arrow) and basal hypercontractility.

**Figure 3:**
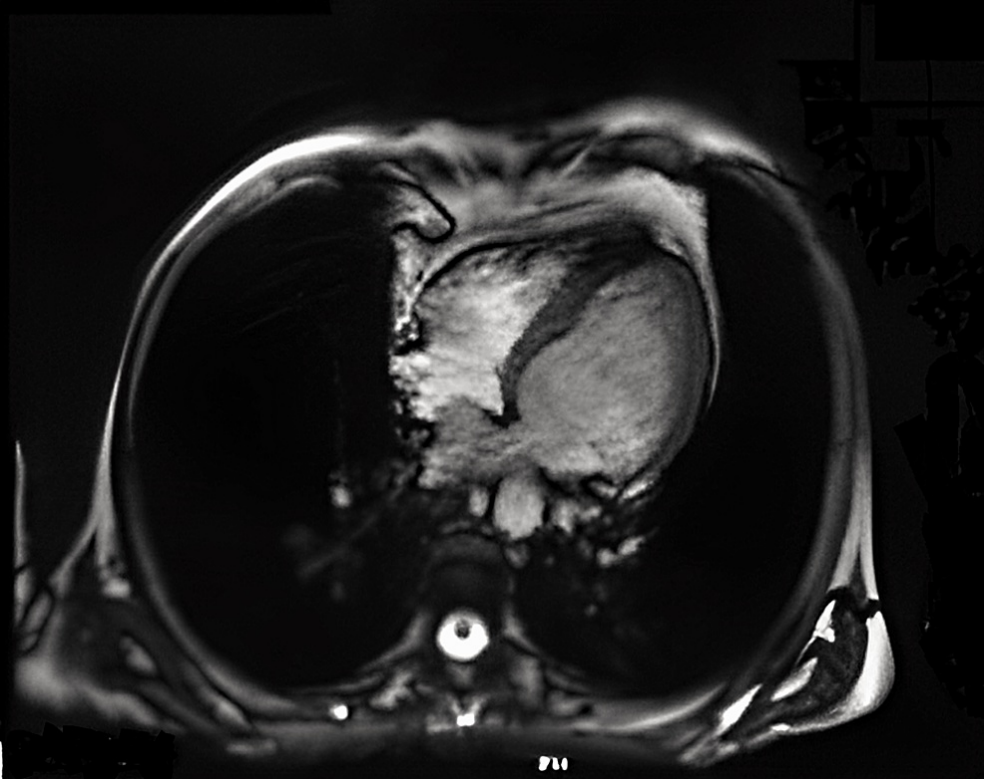
Cardiac MRI Cardiac function in recovery phase (ejection fraction of 45%).

She was admitted to the intermediate care unit for surveillance and continued treatment. An electrocardiogram (ECG) was performed, which, being in sinus rhythm and at a heart rate of 92 bpm, showed the presence of Q waves in the anterior wall, inversion of the T wave in the lateral wall, and an increase in the QTc interval (620 ms) (Figure 1). Transthoracic echocardiography (TTE) showed the systolic left ventricular function to be moderately to severely compromised, with an estimated ejection fraction of about 32%, with mid-distal akinesia and basal hypercontractility, questioning the possibility of the existence of TC (Figure 2). She was promptly medicated with aspirin, beta-blocker, diuretic, and angiotensin-converting enzyme inhibitor. Subsequently, a cardiac MRI was performed, which corroborated these findings but showed the cardiac function was already in the recovery phase, with an increase in ejection fraction of 45% (Figure 3). Cardiac catheterization excluded obstruction of the coronary artery.

**Figure 4:**
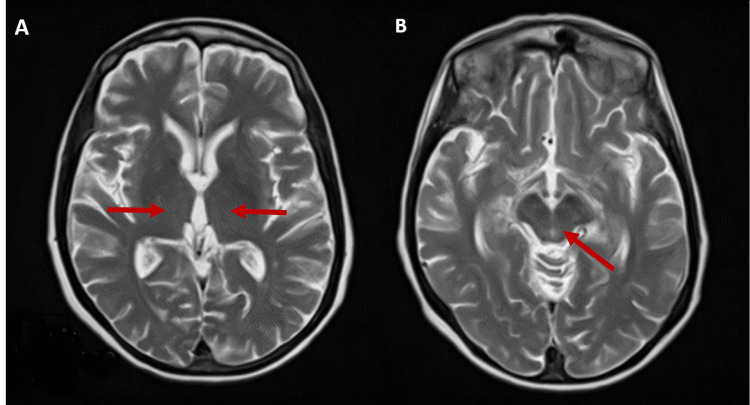
Brain MRI Diffuse cortico-subcortical atrophy, predominantly frontoparietal, with no noticeable signal changes in the brain parenchyma, particularly in the thalamus (A), hypothalamus, mammillary bodies or midbrain (B) (T2-weighted image).

Brain MRI showed marked and diffuse cortico-subcortical atrophy, predominantly cortical and frontoparietal. The T2, fluid-attenuated inversion-recovery (FLAIR), or diffusion-weighted imaging (DWI) sequences did not show noticeable signal changes in the brain parenchyma, particularly in the thalamus, midbrain, and mammillary bodies (namely, the absence of a reduction in their volume) or hypothalamus (Figure 4). The electroencephalogram revealed mild diffuse encephalopathy with slower activity in the right posterior temporal region, and the electromyography supported the diagnosis of predominantly sensory but also motor polyneuropathy.

The patient underwent a psychomotor rehabilitation program during hospitalization. Despite significant neurocognitive improvement with the administration of high doses of thiamine, after a month of treatment, she maintained some degree of apathy and disorientation. She followed simple orders. Muscle strength was preserved and was symmetrical, but she had difficulty discriminating touch in the sensitivity assessment. She needed third-person unilateral support to walk. In the cardiac reassessment at discharge, she showed recovery of the left ventricular systolic function and regional contractility. She was guided to social support at home and to alcohol treatment.

## Discussion

We highlight a case of TC related to alcohol withdrawal in a patient who also seemed to develop WE. Typically, the TC clinical profile is also observed in postmenopausal women, mimicking chest pain and acute coronary syndrome, dyspnea, and palpitations. Often patients state they undergo an acute emotional or physical episode [1]. However, our patient did not experience chest pain or exertional dyspnea, nor did she report that any stressful events had previously occurred. It should be noted that she had a history of chronic alcohol abuse and had suddenly stopped drinking the week before admission, which would have resulted in the symptoms of AWS.

Alcohol is considered a depressant of the central nervous system. The inhibitory tone is increased by modulating the activity of the gamma-aminobutyric acid (GABA) and inhibits the excitatory tone by modulating the activity of the excitatory amino acid (such as glutamate). Adaptation occurs by increasing the number of glutamate receptors in an attempt to maintain a normal state of excitement. Homeostasis is maintained through the existence of ethanol. A sudden interruption will bring up chronic consumption responses with hyperactivity of the central nervous system and an unregulated excess of excitement. Benzodiazepines are the first-line pharmacological treatment of AWS symptoms since they increase the activation of GABAergic neurons [5].

A condition of stress, which can cause an excess of intracellular calcium and improper microvascular function leading to myocyte injury, is one of the most accepted theories explaining the occurrence of TC due to the sympathetic nervous system releasing excess catecholamines [1].

ECG may detect ST-segment elevation, T-wave inversion, and/or QT-prolongation. TC also reveals a high level of cardiac biomarkers, a sign of myocardial disturbance, which is in agreement with ECG revelations. The TC "signature" sign is demonstrated by apical dyskinesia or akinesia with basal hyperkinesia on TTE in 75% of patients [1]. Due to its similarity to myocardial infarction, the first approach needs to be directed to the treatment of ACS. Thus, one criterion for the diagnosis is by excluding coronary artery disease [1]. A β-blocker can be used in TC given the probable elevated condition of catecholamine, though it is not recommendable in the possibility of coronary vasospasm. The administration of an inhibitor of the angiotensin-converting enzyme and a blocker of the angiotensin receptor may also be considered in order to manage the abnormal regional wall motion. Usually, TC has an excellent prognosis, with a 96% recovery rate. Recovery of the LV function may occur in a few days, and full recovery is possible in three to four weeks [1].

The diagnosis of WE is primarily clinical. Classically, the triad consisting of ataxia, ophthalmoplegia, and global confusion is referred to, but these findings are only present in 16-20% of cases [4]. The association of WE with peripheral neuropathy, which usually involves only the lower extremities, is also frequently reported [6].

There are no laboratory tests to diagnose WE with certainty. Blood thiamine assays are not sufficiently sensitive and specific in symptomatic patients. Imaging tests are also not necessary in all patients with suspected WE and, when performed, should not delay treatment [6]. The preferred imaging modality is MRI. The most frequently objectified pathological lesions reported in the literature include an abnormal change in the intensity of bilateral symmetrical lesions in the thalamus and hypothalamus regions as well as the mammillary bodies, periaqueductal region, and fourth ventricle floor. However, it should be noted that this typical pattern of lesions is seen in only 58% of patients, so a normal MRI does not definitely rule out a WE [3].

A deficiency of thiamine results in a Krebs cycle dysfunction (tricarboxylic acid, TCA cycle) and pentose phosphate pathway resulting in brain cytotoxic as well as vasogenic oedema [3]. In alcoholism, thiamine deficiency can be explained by thiamine’s lower uptake or lower absorption rate at the mucosal level and impaired thiamine utilization.

WE may cause irreversible brain damage, resulting in death in almost 20% of cases, and 85% of survivors may suffer from Korsakoff syndrome. The Korsakoff syndrome is a type of retrograde and enterograde amnesia including confabulation connected with lesions of the mammillary bodies and dorsal thalamus and may be found in patients who did not receive treatment for WE [3].

The basis of WE treatment is the prompt administration of thiamine to prevent progression to irreversible deficits, including Korsakoff syndrome. A high level of clinical consideration is required as it is not often easy and may take a long breadth of time to reach a diagnosis. According to the European Federation of Neurological Society, an intravenous infusion of thiamine is recommended, 200 mg diluted with 100 mL of normal saline or 5% dextrose administered over 30 minutes, three times a day until there is improvement in clinical conditions, while British authors recommend 500 mg three times a day for 2 to 3 days and then 250 mg daily until there is an improvement [7]. The administration of thiamine may reduce symptoms, especially if given promptly. Oculomotor abnormalities and some light neurocognitive symptoms, among which drowsiness, confusion, and apathy, respond to this treatment. However, deficits in learning and memory do not respond as well, and many patients remain with residual or permanent Korsakoff amnesia. Balance disorders such as gait ataxia also recover late and sometimes do not go away [3].

This case is consistent with TC, namely due to the characteristic echocardiographic findings, as well as the recovery of the LV function with symptomatic treatment, taking a favorable course. We cannot certainly say that our patient developed a WE as the typical imaging findings were not objective. However, taking into account that the patient had two of the classic triad symptoms, together with the clinical improvement with thiamine's administration, it seems to us to be a very likely hypothesis. Furthermore, the clinical course of WE is compatible with the patient's evolution, in which residual deficits usually remain, mainly in memory, learning, and gait disorders.

## Conclusions

This report described a complex case of TC that was believed to be associated with AWS, with the aggravating factor of concomitantly developing a WE. Proper management of the situation requires a high degree of suspicion from the various entities since the patient's admission. With supportive medical treatment in place, she spontaneously recovered left ventricular function. The patient has improved neurocognitively since thiamine was started, but she remained with residual gait ataxia, apathy, and disorientation.
